# Comparative transcriptomic analysis reveals the oncogenic fusion protein PAX3-FOXO1 globally alters mRNA and miRNA to enhance myoblast invasion

**DOI:** 10.1038/oncsis.2016.53

**Published:** 2016-07-25

**Authors:** J M Loupe, P J Miller, B P Bonner, E C Maggi, J Vijayaraghavan, J S Crabtree, C M Taylor, J Zabaleta, A D Hollenbach

**Affiliations:** 1 Department of Genetics, Louisiana State University Health Sciences Center, New Orleans, LA, USA; 2 Department of Microbiology, Immunology, and Parasitology, Louisiana State University Health Sciences Center, New Orleans, LA, USA; 3 Department of Pediatrics and Stanley S. Scott Cancer Center, Louisiana State University Health Sciences Center, New Orleans, LA, USA

## Abstract

Rhabdomyosarcoma, one of the most common childhood sarcomas, is comprised of two main subtypes, embryonal and alveolar (ARMS). ARMS, the more aggressive subtype, is primarily characterized by the t(2;13)(p35;p14) chromosomal translocation, which fuses two transcription factors, PAX3 and FOXO1 to generate the oncogenic fusion protein PAX3-FOXO1. Patients with PAX3-FOXO1-postitive tumors have a poor prognosis, in part due to the enhanced local invasive capacity of these cells, which leads to the increased metastatic potential for this tumor. Despite this knowledge, little is known about the role that the oncogenic fusion protein has in this increased invasive potential. In this report we use large-scale comparative transcriptomic analyses in physiologically relevant primary myoblasts to demonstrate that the presence of PAX3-FOXO1 is sufficient to alter the expression of 70 mRNA and 27 miRNA in a manner predicted to promote cellular invasion. In contrast the expression of PAX3 alters 60 mRNA and 23 miRNA in a manner predicted to inhibit invasion. We demonstrate that these alterations in mRNA and miRNA translate into changes in the invasive potential of primary myoblasts with PAX3-FOXO1 increasing invasion nearly 2-fold while PAX3 decreases invasion nearly 4-fold. Taken together, these results allow us to build off of previous reports and develop a more expansive molecular model by which the presence of PAX3-FOXO1 alters global gene regulatory networks to enhance the local invasiveness of cells. Further, the global nature of our observed changes highlights the fact that instead of focusing on a single-gene target, we must develop multi-faceted treatment regimens targeting multiple genes of a single oncogenic phenotype or multiple genes that target different oncogenic phenotypes for tumor progression.

## Introduction

Rhabdomyosarcoma (RMS), which accounts for nearly half of childhood soft tissue sarcomas, is comprised of two main subtypes: embryonal rhabdomyosarcoma and alveolar (ARMS), each defined by its unique histology, clinical presentation and prognosis.^1^ ARMS, the more aggressive subtype, is primarily defined by the t(2;13)(p35;p14) chromosomal translocation,^2^ which generates the oncogenic fusion protein PAX3-FOXO1.^3, 4^ PAX3-FOXO1 has altered molecular activities relative to wild-type PAX3, including being a more potent transcriptional activator,^5^ being unresponsive to normal PAX3 co-regulators^6^ and having greater post-translational stability upon the induction of myogenic differentiation.^7^ These aberrant molecular activities are believed to contribute to altered gene regulation, including the activation of genes not normally regulated by PAX3^8^ and increased expression of other genes relative to PAX3,^9, 10^ which taken together is believed to contribute to ARMS tumor phenotypes.^11^

Patients diagnosed with PAX3-FOXO1-positive ARMS have a 4-year survival rate of 8%.^12^ This poor prognosis stems in part from these tumor cells having a higher incidence of localized invasion,^12^ which may then lead to heightened aggressiveness and an increased propensity for metastasis. The presence of PAX3-FOXO1 is known to enhance the invasive potential of cells,^13^ possibly through its ability to alter the expression of multifunctional genes that contribute, in part to invasion in other tumor types, including MET,^10^ FGFR4,^14^ IGF2^15^ and IGFBP5.^15^ Despite these circumstantial correlations, to date only a single report demonstrates that the PAX3-FOXO1 altered expression of a gene, the cannabinoid receptor 1, directly contributes to the invasive capacity in ARMS.^16^ However, these results were derived from the expression of the oncogenic fusion protein in established tumor cell lines^13^ or in primary myoblasts that genetically contained compensatory oncogenic mutations.^16^ Further, these reports either did not examine altered gene expression^13^ or focused their study on changes in the expression of a single gene.^16^ While these reports are noteworthy and of importance, they provide little information to describe how the expression of PAX3-FOXO1 in the absence of any other compensatory mutations globally alters mRNA expression patterns to contribute to invasion. Further, to date no studies have directly examined how the presence of PAX3-FOXO1 affects microRNA (miRNA) expression and how these changes contribute to the invasive capacity of myoblasts.

In this study we utilized physiologically relevant wild-type primary myoblasts along with large-scale comparative transcriptomic analyses to examine how the expression of PAX3-FOXO1 or PAX3 alters global mRNA and miRNA expression profiles and how these changes contribute to the invasive potential of these cells. We report here that the expression of the oncogenic fusion protein is sufficient to alter the expression of 70 mRNA and 27 miRNA in such a way that would be expected to promote cellular invasion. In contrast, the expression of PAX3 elicits mRNA and miRNA expression changes that would be expected to inhibit cellular invasion. We found that these mRNA and miRNA changes translate into biological effects, with the expression of PAX3-FOXO1 enhancing and the expression of PAX3 inhibiting primary myoblast invasion. Taken together, these results provide a more expansive picture to describe the increased localized invasion seen with t(2;13)(q35;q14) positive ARMS tumors, and describes how the presence of PAX3-FOXO1 may contribute to higher levels of metastasis in these patients.

## Results and Discussion

**Figure 1:**
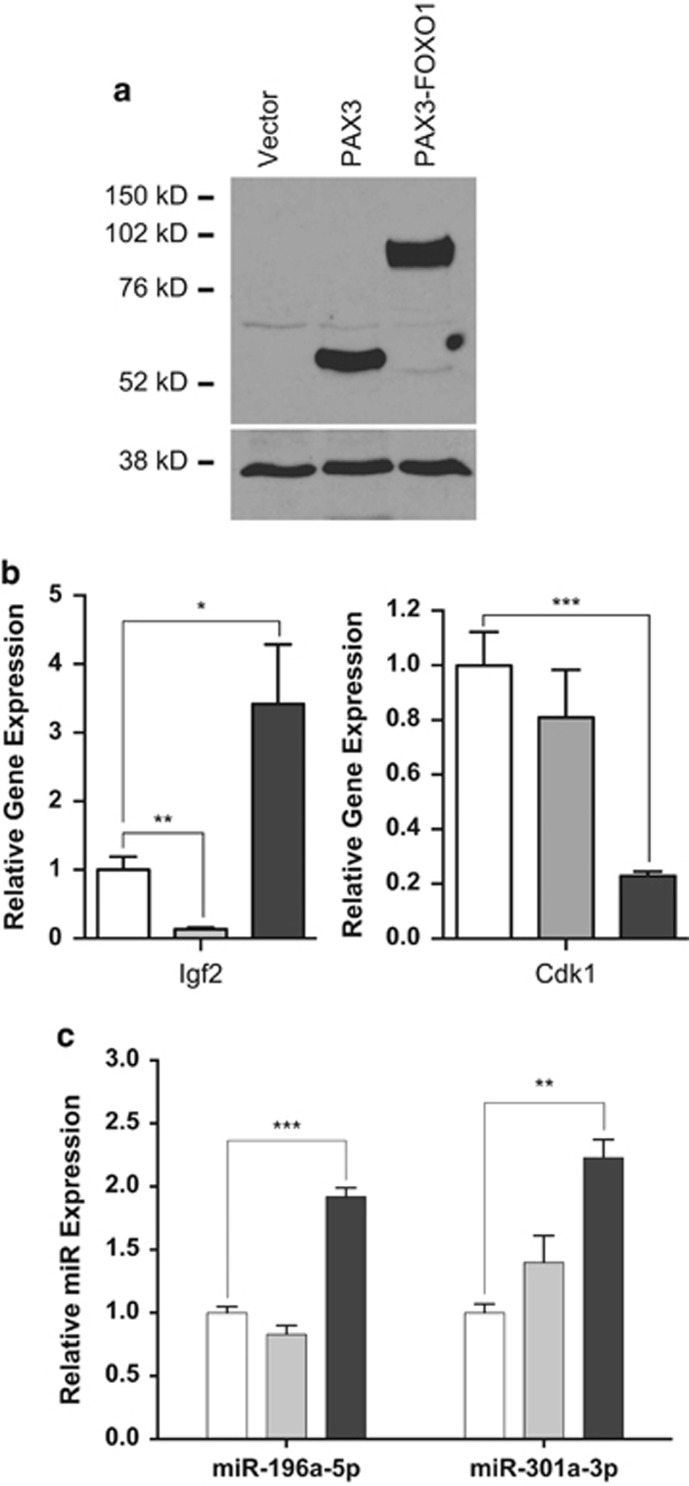
Protein expression (**a**) and quantitative RT–PCR analyses for (**b**) select mRNA and (**c**) select miRNA. Mouse primary myoblasts were isolated from 2- to 4-day-old C57/Bl6 mice as previously described.^53^ Cells were grown as previously described^7, 17, 18, 19, 53^ and were passage-matched to prevent possible differences due to passage conditions. Mouse primary myoblasts were stably transduced as previously described^6, 53^ with the MSCV-IRES-puromycin empty vector, vector containing FLAG epitope-tagged Pax3 (FLAG-Pax3) or FLAG-PAX3-FOXO1. Three days post transduction, cells were selected using puromycin, as previously described.^19^ The stably transduced cells were harvested and pooled from three independent transductions to create a single population that express each construct. (**a**) Total cell extracts made, as previously described.^17, 18, 19, 53^ Equal amounts of total cell lysates (12 μg) were separated by 8% SDS–PAGE and analyzed by western blot analysis using antibodies specific for Pax3,^54^ as previously described.^18, 19^ (**b**,**c**) Total RNA was isolated from the stably transduced proliferating primary myoblasts (empty vector (white bars), PAX3 (gray bars) or PAX3-FOXO1 (black bars)) using the miRNeasy mini kit (Qiagen, Madison, WI, USA), allowing for the isolation of RNA <30 bp in length, according to the manufacturer's specifications. Equal amounts of total RNA (100 ng) were used for cDNA synthesis using the iScript cDNA synthesis kit (Bio-Rad, Hercules, CA, USA) for mRNA (**b**) or the Taqman miRNA reverse transcription kit (Applied Biosystems, Foster City, CA, USA) for miRNA. (**c**) The qRT–PCR was performed on the resulting cDNA using the CFX96 Touch Real-Time PCR Detection System (Bio-Rad) using commercially available primer/probe sets and the Applied Biosystems Universal Master Mix (Applied Biosystems), according to the manufacturer's specifications. All results were normalized for GAPDH (mRNA) or the U6 small nuclear RNA (miRNA) and reported as fold expression relative to the results obtained for cells stably transduced with the empty vector. In all cases, analyses were performed comparing each sample with the empty vector control (*P=0.009, **P=0.001, ***P=0.0001).

To understand how PAX3-FOXO1 affects global mRNA and miRNA expression, we stably transduced passage-matched wild-type mouse primary myoblasts with the MSCV-IRES-puromycin retroviral vector (negative control), or the same retroviral vector expressing FLAG epitope-tagged PAX3 (FLAG-PAX3) or FLAG-PAX3-FOXO1, a tag previously shown to not affect Pax3 or Pax3-FOXO1 function.^6, 17^ The puromycin selected cells were harvested from three independent transductions and pooled, resulting in a single mixed population for each individual construct, which removes the potential for variability that may occur from clonal effects. The level of PAX3-FOXO1 expression is equivalent to the level of expression of the fusion protein in ARMS tumor cell lines (Figure 1 and Dietz *et al.*^18, 19^) and is therefore directly relevant to the role of the oncogenic fusion protein in ARMS. This model allows us to use a physiologically relevant cell system in the absence of any complimentary transforming mutations to determine the specific effects of PAX3-FOXO1 on oncogenic phenotypes.

We performed mRNA and miRNA deep-sequencing analyses on total RNA isolated from three independent growths of stably transduced cells and utilized the resulting data to perform comparative transcriptomic analyses to understand how each protein alters expression profiles to exert their effects on the invasive capacity of cells. For both the mRNA and miRNA analyses the data used for subsequent studies were limited to (1) those genes or miRNA displaying statistically significant differences (*P*<0.05, as determined by the Galaxy Cuffdiff program (mRNA) or miRNAKey (miRNA)), (2) transcripts whose expression was present in both data sets being analyzed to rule out potential artifactual differences resulting from depth of read and (3) transcripts or miRNA that exhibited at least 2-fold differences in expression either up or downregulated.

We found a total of 480 mRNA whose expression changed in a PAX3-FOXO1-dependent manner (276 downregulated and 204 upregulated) relative to the empty vector negative control (data not shown). We performed a PubMed search on each of the 480 mRNA, using the gene name and the search term ‘invasion' to determine if they were experimentally proven to contribute to cellular invasion. We found that 70 of the 480 altered genes (14.5%) are involved in regulating the invasive capacity of cells (Table 1). Forty-three of these genes have literature evidence demonstrating their role in promoting cellular invasion, with these altered genes being split nearly equally between being upregulated (19/43; 44%) or downregulated (24/43; 56%) in a PAX3-FOXO1-dependent manner. In a similar manner, 27 genes have literature evidence to support their role in inhibiting cellular invasion, with 21 of these genes (nearly 80%) being downregulated in a PAX3-FOXO1-dependent manner. Finally, 17 of the 70 differentially expressed genes (nearly 25%) contain PAX3-FOXO1 binding sites in their proximal promoters, as previously described^20^ (Table 1, c), four of these genes were previously demonstrated to be regulated by PAX3-FOXO1, including cannabinoid receptor 1,^16^ FGFR4,^20^ IGF2^21^ and IGFBP5^15^ (Table 1, b), and 21 of the 70 (30%) genes have altered gene expression levels consistent with changes seen in human tumor samples^22, 23, 24, 25^ (Table 1, a).

An initial examination of the distribution of mRNA whose levels are altered upon the expression of the fusion protein would suggest that PAX3-FOXO1 would primarily exert its invasive effect^13^ by decreasing the expression of genes important for inhibiting invasion. However, it is interesting to note that although only 44% of the genes that promote invasion are upregulated, nearly half of these 19 upregulated genes (8/19–42%) are increased >6-fold, including the previously reported cannabinoid receptor^16^ (cannabinoid receptor 1–6.92-fold), with the top four genes being upregulated >20-fold. Therefore, this data suggest that PAX3-FOXO1 exerts its effects on invasive capacity by not only decreasing the expression of a large number of inhibitory genes, but by simultaneously greatly increasing the expression of key genes that promote invasion, including genes that encode for proteins involved in cytoskeletal organization (CAP6–33.45-fold), cadherins (CDH6–23.23-fold), extracellular matrix metalloproteases (ADAMTS1–21.98-fold) and cell adhesion proteins (MSLN – 20.93-fold).

A similar analysis found 399 mRNA change in a PAX3-dependent manner (276 downregulated and 123 upregulated) relative to the empty vector negative control (data not shown). A similar PubMed search revealed that 60 of the 399 genes (15%) are involved with regulating invasion (Table 1). Thirty-eight of these genes have a demonstrated role in promoting invasion, with a majority of these genes (25/38; 66%) being downregulated. Further, 22 mRNA were demonstrated to inhibit invasion, with 6 of these genes being upregulated and 16 being downregulated. Finally, four of the differentially expressed genes were demonstrated in the literature to be directly regulated by PAX3, including Ahr,^26^ IGF1R,^20^ EPHA2^27^ and MET^28^ (Table 1, b). Although a smaller number of these inhibitory genes are upregulated, one of them is upregulated >15-fold (metallopeptidase Mme—15.17-fold). In contrast to the results seen with PAX3-FOXO1, these data suggest that PAX3 would be expected to inhibit invasive capacity, primarily through the downregulation of genes that promote this biological event.

A comparative transcriptomic analysis of the miRNA data identified a total of 84 miRNAs whose expression changed in a PAX3-FOXO1-dependent manner (46 downregulated and 38 upregulated) relative to the empty vector negative control (data not shown). A PubMed search of each of the individual 84 miRNA, using the miRNA name and the search term ‘invasion', indicated that 10 of these miRNA promote cellular invasion (Table 2). Of these miRNA, 9/10 (90%) have an increased PAX3-FOXO1-dependent expression with the top two being increased >20-fold. In a similar manner, 17 miRNA are important for inhibiting cellular invasion, of which 16/17 (94%) are downregulated, with the top four being downregulated >12-fold. In conjunction with the results of our PubMed search, which also described the target genes responsible for the invasive effect of the miRNA, we used miRTarBase^29^ to identify known target genes whose biological function may contribute to an invasive phenotype, with validation on miRTarBase by at least two independent experimental methods. Interestingly, only a small number of the altered miRNA have the expected inverse correlation to our observed changes in mRNA expression (miR-222/miR-221 and TIMP2, and miR-362 and CD82).

A similar analysis determined a total of 58 miRNA whose expression changed upon the expression of PAX3 (25 downregulated and 33 upregulated) relative to the empty vector negative control (data not shown). Of these genes, a PubMed search determined that 7 are important for promoting while 16 inhibit cellular invasion. Of those miRNA, 5/7 (71%) that promote invasion are decreased whereas 10/16 (63%) that inhibit invasion are increased, with the top inhibitory miRNA being increased >15-fold. Finally, for both PAX3-FOXO1- and PAX3-dependent miRNA changes, the literature provides direct evidence for the genes they target in order to exert their effects on invasion (Table 2). As seen with PAX3-FOXO1 changes, only one of the PAX3-altered miRNA has the expected inverse correlation to mRNA expression (miR-206 and MET, Tables 1 and 2). Interestingly, three sets of miRNA are present as clusters in the mouse genome and have similar changes in expression. These include miRNA 222 and 221, which are upregulated to a similar extent by PAX3-FOXO1 while being downregulated to a similar extent by PAX3 (Table 2, a); miRNA 362 and 532, which are downregulated to a similar extent by PAX3-FOXO1 but are unaffected by PAX3 (Table 2, b); and miRNA 133b and 206, which are unaffected by PAX3-FOXO1 are upregulated to a similar extent by PAX3 (Table 2, c).

To validate the observed changes, we performed a quantitative RT–PCR analysis on a subset of mRNA and miRNA. We tested IGF2, which was reported to promote cellular invasion in a variety of tumors,^30, 31, 32, 33^ and CDK1, which has alternative roles in inhibiting cellular invasion in different tumor models.^34, 35, 36, 37, 38^ We also examined two miRNA (miR-196a-5p and miR301a-3p), both demonstrated to promote cellular invasion.^39, 40, 41, 42, 43, 44^ We observed quantitative and significant changes in expression for all of the mRNA and miRNA examined that are consistent with our mRNA deep-sequencing results (Figure 1).

**Figure 2:**
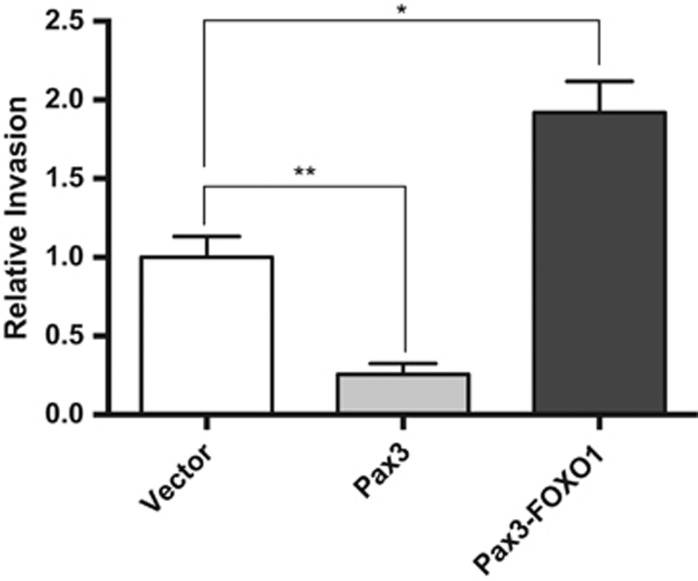
Pax3-FOXO1 promotes whereas Pax3 inhibits primary myoblast invasive capacity. Invasive capacity was determined using stably transduced proliferating primary myoblasts (empty vector (white bars), PAX3 (gray bars) or PAX3-FOXO1 (black bars)) using the BD Biocoat Tumor Invasion System (Becton Dickinson, Franklin Lakes, NJ, USA). About 50 000 cells suspended in proliferation media were added to the insert plate with proliferation media supplemented with hepatocyte growth factor (hHGF, PeproTech, Rocky Hill, NJ, USA) at 25 ng/ml being used as the chemoattractant. After 24 h of incubation, the insert system was transferred to a second 24-well plate containing calcein AM in Hank's balanced salt solution (HBSS) that enabled the fluorescent labeling of cells that invaded through the Matrigel matrix. Fluorescence of the invaded cells was read at wavelengths of 494/517 (Ex/Em) using a Synergy HT multi-well microplate reader (BioTek, Winooski, VT, USA). *P*-values were computed using non-parametric one-way ANOVA analysis comparing all samples with results obtained, with cells expressing empty vector (*P=0.03, **P=0.001). ANOVA, analysis of variance.

Our comparative transcriptomic results suggest that the mRNA and miRNA changes induced by the oncogenic fusion protein would be predicted to promote cellular invasion, whereas those changes that occur in a PAX3-dependent manner should inhibit the invasive capacity of cells. Therefore, we used a standard invasion assay to determine whether our observed mRNA and miRNA changes translate into experimental differences in the invasive potential of primary myoblasts. Consistent with our mRNA and miRNA changes, we observed a nearly 2-fold increase in primary myoblasts expressing PAX3-FOXO1, consistent with the previous reports,^16^ whereas cells expressing PAX3 had a nearly 4-fold decrease in invasive potential (Figure 2).

Taken together, our results build off of previous work, in which a single gene was examined,^16^ and show that the sole expression of PAX3-FOXO1 in the absence of any complimentary genetic mutations is capable of globally altering mRNA and miRNA levels to promote the invasive capacity of primary myoblasts. Further, this is the first study to examine how the oncogenic fusion protein alters miRNA levels, which combined with our global mRNA results allow us to develop a more expansive picture of the underlying regulatory mechanisms by which the expression of PAX3-FOXO1 promotes invasion.

In this regulatory mechanism, the somatic and random acquisition of the chromosomal translocation creates the fusion protein, which alters, either directly or indirectly, the expression of mRNA important for enhancing cellular invasion. PAX3-FOXO1 achieves this by both decreasing the expression of genes that inhibit invasion (80% of downregulated genes) while also greatly increasing the expression of nearly half of the altered genes that promote invasion (Table 1). However, our results demonstrate that miRNAs, which post-transcriptionally ‘fine tune' gene expression, also have a significant role, as nearly all of the increased miRNAs promote invasion and a majority of the decreased miRNAs inhibit invasion. Further, a closer inspection of the data reveals that although some of the miRNA changes have an inverse correlation to target genes present in our results, there are only three miRNA (miR-221, miR-222 and miR-362) that have such a correlation with their target genes (TIMP2 and CD82, respectively). Therefore, instead of post-transcriptionally contributing to our observed mRNA changes, the altered miRNA target a different set of genes important for invasive capacity, thereby greatly increasing the number of affected genes.

The global nature of the mRNA and miRNA expression changes that result upon the sole expression of PAX3-FOXO1 provide a basis for how it may be necessary to rethink approaches to the development of therapies for ARMS. At present, many developmental ARMS therapies focus on attacking a single gene or pathway mechanistically located downstream of the fusion protein. However, given that the expression of PAX3-FOXO1 alters the expression of 70 different genes and 27 different miRNAs to affect the invasive potential of cells, it is not too surprising that such focused and targeted therapies are not proving effective in Phase I or Phase II clinical trials for ARMS.^45, 46, 47, 48^ It is conceivable that the loss of a single gene through these targeted and focused therapies could easily be compensated through the changes in nearly 100 other affected genes, thereby negating the effects of the treatment.

Work in the past few years identified multiple aspects of the invasive process as potential targets for therapy development. Along these lines we propose developing a multi-faceted regimen that targets several of these processes, targets that include tumor-promoting genes we found to be the most highly upregulated in our study (Table 1). These processes include cytoskeletal remodeling, which is mediated in part by the intracellular signaling cysteine proteases calpains^49^ (Capn6 is upregulated 33-fold in our study), cellular adhesion mediated by such molecules as mesothelin^50^ (Msln is upregulated 21-fold in our study) and matrix metalloproteases,^51^ in particular the Adamts family of proteases^52^ (Adamts1 is upregulated 22-fold and Adamts5 is upregulated 7.5-fold in our study). A regimen that minimally targets these three processes would inhibit the necessary biological events required for invasion and metastasis. Alternatively, inhibiting one of these events (for example, matrix metalloproteases) could serve as one arm of a novel multi-faceted regimen for the treatment of ARMS, a regiment that also targets other ARMS molecular processes such as inhibiting phosphorylation of PAX3-FOXO1,^17^ attacking aneuploid cells and preventing enhanced proliferation.

## Figures and Tables

**Table 1:** Altered mRNA expression important for tumor cell invasion

*Gene*	*Gene function*	*V vs PF*	*V vs P3*
*mRNA that promote tumor invasion*			
*Capn6*	Cytoskeletal organization	33.45	
*Cdh6*	Type II cadherin, development	23.23	9.17
a*Adamts1*	Metalloprotease	21.98	5.65
*Msln*	Cell adhesion; overexpressed in cancers	20.93	48.60
a*Adamts5*	Peptidase; aggrecanase to cleave aggrecan	7.68	
a,^b^*Cnr1*	G-protein signaling	6.92	
*Hoxb9*	Transcriptional activator increased in cancers	6.34	
a,b,^c^*Fgfr4*	FGF receptor	6.12	
a,^b^*Igf2*	Growth factor	5.37	−13.18
c*Plxna2*	Semaphorin co-receptors	3.32	
*Erbb3*	EGFR receptor tyrosine kinases	3.10	−4.04
*Klf5*	Possible transcription factor	2.84	
*Pbx3*	Transcriptional activator	2.59	
*Stat3*	Expression of genes in response to cell stimuli	2.58	
c*Sulf2*	Remove 6-O-sulfate groups from heparan sulfate	2.52	
c*Lamc1*	Mediate attachment, migration-interacting extracellular matrix	2.45	
*Pkp2*	Linking cadherins to intermediate filaments	2.40	
*Cdc25b*	Activates the cyclin-dependent kinase CDC2	2.20	
a*Ncam1*	Cell adhesion; cell-to-cell interactions	2.00	−2.95
*Tpm3*	Provide stability to actin filaments	−2.08	
*Fscn1*	Cell migration, motility and adhesion	−2.09	
*Mdm2*	E3 ubiquitin-protein ligase	−2.12	
a*Adam19*	Matrix metalloproteinase	−2.15	−3.08
a*Jag2*	Ligand that activates Notch	−2.17	−4.05
*Cttn*	Adherins and cytoskeleton	−2.23	
*Arpc5*	Control of actin polymerization	−2.34	
a,^c^*Abi1*	Mediates signal transduction from Ras to Rac	−2.42	
a*Jak1*	Cell signal transduction	−2.45	
*Mmp14*	Metalloproteinase	−2.58	
*Ifitm1*	Implicated in cell adhesion	−2.68	−2.88
a*Elk3*	Activated by signal-induced phosphorylation	−2.74	
*Vim*	Cytoskeletal protein	−2.90	
*Id1*	Inhibits the DNA-binding transcription factors	−3.00	
*Pak1*	Cell motility and morphology	−3.21	
a*Cyr61*	Promotes the adhesion of endothelial cells	−3.28	
a*Dusp1*	Cellular response to environmental stress	−3.62	
a*Lasp1*	Binds to the actin cytoskeleton	−3.91	
*Ntn4*	Protein related to laminins	−4.93	
*Etv4*	Transcriptional activator	−5.12	
a,^c^*Flnb*	Filamin; repair vascular injuries	−5.82	
*Axl*	Transduces signals from the extracellular matrix	−6.04	
a*Igfbp2*	Inhibits IGF-mediated growth	−8.64	
*Cxcl12*	Chemotaxis; embryonic development	−19.80	−14.90
b*Ahr*	Ligand-activated transcriptional activator		11.13
*Egfr*	Receptor for members of EGF family		4.82
*Eps8*	Functions as part of the EGFR pathway		3.59
*Sema3e*	Axon guidance; Semaphorins		3.43
*Galnt2*	Oligosaccharide biosynthesis		2.64
*Sparc*	Involved in ECM synthesis		2.44
*Ghr*	Transmembrane receptor for growth hormone		2.28
*Prdx1*	Antioxidant protective		2.27
*Emp3*	Involved in proliferation and cell–cell interactions		2.04
*Rnf11*	Transcriptional activator		−2.01
*Myo5a*	Cytoplasmic vesicle transport and anchorage		−2.09
b,^c^*Igf1r*	Critical role in transformation events		−2.13
*Hes6*	Promotes cell differentiation		−2.17
*Abl2*	Non-receptor tyrosine protein kinases		−2.21
*Peak1*	Role in cell spreading and migration		−2.24
*Zkscan3*	Transcriptional regulator		−2.35
*Tppp3*	Tubulin and has microtubule-bundling activity		−2.45
*St3gal1*	Transfer of sialic acid to substrates		−2.47
*Bach1*	Transcription factor		−2.50
b*Epha2*	Ephrin receptor subfamily		−2.53
*Notch1*	Developmental processes by controlling cell fate		−3.07
*Jun*	Transcriptional activator		−3.12
*Kdm5b*	Histone demethylase; transcriptional corepressor		−3.17
c*Sema6a*	Cell surface receptor for cell–cell signaling		−3.46
b,^c^*Met*	Hepatocyte growth factor receptor		−3.73
*Nuak1*	Multifunctional kinase		−3.87
*Serpine2*	Inhibit serine proteases		−4.99

*mRNA that inhibit tumor invasion*			
a,^b^*Igfbp5*	Alter the interaction of IGFs with receptor	8.28	−5.68
c*Spry1*	Antagonist of FGF pathways	7.39	
*Serpinb1*	Proteinase inhibitor	6.48	
a*Dcx*	Bind microtubules	5.16	
*Col4a2*	Inhibitor of angiogenesis and tumor growth	2.83	
*Cd82*	Metastasis suppressor	2.62	−2.37
*Spry2*	Inhibitory effect on growth factor signaling	−2.10	
*Deptor*	Negative regulator of the mTORC1 signaling	−2.12	
c*Cited2*	Inhibits transactivation of HIF1A-induced genes	−2.26	−4.02
*Actn1*	Nonmuscle, cytoskeletal, alpha actinin isoform	−2.32	
*Flna*	Remodeling the cytoskeleton	−2.43	
*Lpp*	Involved in cell–cell adhesion and cell motility	−2.62	
c*Dlg5*	Transmission of signals to the cytoskeleton	−2.66	
a*Timp2*	Inhibitors of the matrix metalloproteinases	−2.68	
*Tagln*	Actin crosslinking/gelling protein	−2.70	−3.56
c*App*	Transcriptional activator	−2.88	
*Cdk1*	Cell cycle regulatory kinase	−3.01	
*Creb3l1*	Transcriptional activator	−3.04	
*Dusp4*	Phosphatase; negatively regulate (MAP) kinases	−3.08	
a*Id3*	Inhibits the DNA-binding transcription factors	−3.13	
*Wisp1*	Downstream in the WNT1 signaling pathway	−3.32	−2.36
*Rgs16*	Inhibits signal transduction	−3.78	−4.93
c*Tns3*	Cell migration and bone development	−4.18	−4.30
*Filip1l*	Regulator of the anti-angiogenic activity	−5.68	−2.21
*Sox4*	Regulation of embryonic development	−6.48	−2.91
a*Akap12*	Scaffold protein in signal transduction	−7.98	−3.87
*Fstl1*	Modulate action of growth factors	−8.56	−2.16
c*Mme*	Metallopeptidase		15.17
c*Gprc5a*	Development, cellular growth and differentiation		3.67
*Nefl*	Intracellular transport to axons and dendrites		3.31
*Dusp6*	Negatively regulate (MAP) kinases		2.93
*Galnt7*	GalNAc transferase 7		2.48
*Adam9*	Biological processes: cell–cell/matrix interactions		2.44
*Flnc*	Crosslink actin filaments		−2.15
*Rhob*	Cell adhesion and growth factor signaling		−2.58
*Mtss1*	Actin bundling		−2.77
*Igfbp3*	Bind and inhibit IGF (affect growth)		−3.33
c*Dyrk2*	Cellular growth and/or development		−3.36

Abbreviations: FGF, fibroblast growth factors; EGFR, epidermal growth factor receptor; IGF, insulin-like growth factor.

Total RNA was isolated using the miRNeasy mini kit (Qiagen), allowing for the isolation of RNA <30 bp in length, according to the manufacturer's specifications. Poly-A+ mRNA was isolated from 4 μg total RNA, to generate the cDNA libraries, using the Illumina sample preparation kit according to the manufacturer's specifications (Illumina, San Diego, CA, USA). The cDNA libraries were provided a unique index identifier, allowing the clustering of several samples into a single sequencing lane, and deep-sequencing analyses were performed in triplicate from three independent cell growth, RNA isolation and cDNA library constructions. The raw data were groomed and trimmed for quality of the read using online Galaxy analysis (https://usegalaxy.org), resulting in 40–41 high-quality base pair reads for each sequence with between 4–6 million independent reads for each sample. The sequences were mapped to the mouse genome using Tophat analysis, transcripts were assembled using the Cufflinks program, and individual replicates were merged into a single file using Cuffmerge. The resulting transcript reads were normalized using Fragments Per Kilobase of transcript per Million mapped reads analysis, which normalizes each identified sequence for the length of the identified transcript and the volume of the total read yield from each run. Differential expression was determined from these normalized values comparing vector versus Pax3-FOXO1 (V vs PF) or vector versus Pax3 (V vs P3) using the Cuffdiff program, which not only compares differential expression of the merged files between sets but also utilizes the sequence results from the three independent determinations within each set to assign statistical significance to the differential expression.

a Indicates genes with similar trends in expression changes in human tumor samples.^22, 23, 24, 25^

b Indicates genes demonstrated in the literature to be direct targets of PAX3 or PAX3-FOXO1.^15, 16, 20, 21^

c Indicates genes with known PAX3-FOXO1 binding sites in their promoter.^20^

**Table 2:** Altered miRNA expression important for tumor cell invasion

*miRNA*	*Gene target*	*Gene function*	*V vs PF*	*V vs P3*
*miRNA that promote tumor cell invasion*				
615-3p			+30.45	+5.01
196a-5p	HOXA5 ING5	Developmental transcription factor Suppresses growth and invasion	+24.39	—
30d-3p	GALNT7	Glycopeptide transferase	+3.39	—
301a-3p	SMAD4 TXNIP BBC3 PTEN COL2A1 RUNX3 TGFBR2 SOCS6	Signal transduction activator Suppressor of tumor cell growth Pro-apoptotic protein Tumor suppressor protein Collagen 2 alpha 1 Transcriptional tumor suppressor TGF beta receptor Suppressor of cytokine signaling	+3.07	—
a222-3p	TIMP2 TIMP3	Metallopeptidase inhibitor Metallopeptidase inhibitor	+2.78	−2.52
a221-5p	RECK TIMP2 MMP3 MMP9 PTEN TIMP3	Negatively regulates metalloproteinases Metallopeptidase inhibitor Matrix metalloproteinase Matrix metalloproteinase Tumor suppressor Metallopeptidase inhibitor	+2.63	—
155-5p			+2.18	−2.53
a221-3p	MMP3 MMP9 PTEN TIMP3	Matrix metalloproteinase Matrix metalloproteinase Tumor suppressor Metallopeptidase inhibitor	+2.10	−3.31
183-5p	ITGB1 SOCS6 PDCD4	Integrin—cell adhesion receptor Cytokine signal transduction regulator Inhibit translation—tumor suppressor	+2.05	——
b362-3p	CD82	Metastasis suppressor protein	−3.67	—
Let-7g-5p	GAB2 FN1	Signaling adaptor protein Cell surface adhesion molecule	—	−2.18
28a-5p	CCND1 HOXB3	Cyclin D1 Developmental transcription factor	—	−2.10
23b-3p	PTEN ATG12 HMGB2	Tumor suppressor Regulates autophagy Architectural transcription factor	—	+2.19

*mRNA that inhibit tumor formation*				
1a-3p	TAGLN2	Unknown function	−18.50	—
145a-5p	HIF-2 alpha EGFR OCT4 MUC1 MYC D52	Hypoxia-induced transcription factor Growth factor receptor Developmental transcription factor Cell adhesion molecule Growth-related transcription factor Unknown—overexpressed in cancer cells	−16.76	—
133a-5p	TAGLN2 LASP1 FSCN MMP14	Unknown function Actin-binding protein Actin-binding protein Matrix metalloproteinase	−16.76	—
335-5p	SP1	Transcriptional regulator	−12.50	+2.95
b532-5p	CXCL2	Regulatory chemokine	−4.67	—
148a-3p	S1PR1	Receptor to regulate adhesion	−4.41	−2.98
133a-3p	TAGLN2 LASP1 FSCN MMP14	Unknown function Actin-binding protein Actin-binding protein Matrix metalloproteinase	−3.72	—
148b-3p	WNT NRP1	Developmental ligand Membrane-bound signaling protein	−3.44	—
19a-3p	FRA1	FOS family member	−2.87	—
29a-3p	HSP47 LAMC2 ITGA6	Serine proteinase inhibitor Extracellular matrix glycoprotein Integrin—cell adhesion receptor	−2.65	—
34b-5p			−2.60	—
149-3p	FOXM1 RAP1a RAP1b	Transcription factor Adhesion signaling protein Adhesion signaling protein	−2.34	−7.04
c133b-5p	MMP14	Matrix metalloproteinase	−2.26	—
30d-5p	CCNE2	Cyclin E2	−2.22	—
574-3p	RAC1 EGFR EP300	GTPase-signaling molecule Growth factor receptor Histone acetyltransferase—chromatin	−2.07	—
339-5p	NACC1 BCL6 MDM2	Transcriptional corepressor Transcriptional corepressor Regulator of p53 stability	−1.93	+2.19
338-3p	SMO MMP9 PREX2a ZEB2 MACC1	G-protein coupled receptor Matrix metalloproteinase Guanine nucleotide exchange factor Transcriptional repressor Transcriptional activator	+2.09	+15.27
c133b-3p	FSCN1 MMP9	Actin-binding protein Matrix metalloproteinase	—	+5.74
c206-3p	MET Cdc42 NOTCH3	Growth factor receptor Regulates actin polymerization Developmental receptor	—	+4.49
582-5p	RAB27a PGGT1B LRRK2 DIXDC1	Membrane-bound GTPase Geranylgeranyl transferase enzyme Leucine-rich repeat kinase Positive regulator of Wnt signaling	—	+4.41
345-5p	BAG3	Inhibits HSP chaperone activity	—	+3.09
c206-5p	MET Cdc42 NOTCH3	Growth factor receptor Regulates actin polymerization Developmental receptor	—	+2.31
486-5p	ARHGAP5 PIK3R1 OLFM4	Rho GTPase-activating protein PI3K regulatory subunit Extracellular matrix glycoprotein	—	+2.09
31-3p			+4.19	+2.05
34c-3p	PAC1 MARCKS eIF4E	Adenylate cyclase-activating receptor F-actin crosslinking protein Translation elongation factor	+3.38	+3.33
615-5p	AKT2 IGF2	Ser/Thr protein kinase Growth factor ligand	+34.90	−2.53
193-3p	ERBB4 S6K2	Receptor tyrosine growth factor receptor Ribosomal kinase	+3.26	—
181c-3p	SMAD7	Negatively regulates TGF beta signaling	—	−3.77
30a-5p	ITGB3 NCAM SEPT7 MTDH	Integrin—cell-adhesion receptor Cellular-adhesion molecule Cytoskeletal GTPase—actin organization Activates NFkB	—	−2.50
30c-2-3p	TRADD CCNE1	Mediates apoptosis and NFkB signaling Cyclin E1	—	−2.32

Abbreviation: HSP, heat shock proteins.

Total RNA was isolated using the miRNeasy mini kit (Qiagen), allowing for the isolation of RNA <30 bp in length, according to the manufacturer's specifications. miRNA was isolated from 4 μg total RNA to generate the cDNA libraries, using the Illumina sample preparation kits according to the manufacturer's specifications. The cDNA libraries were provided a unique index identifier, allowing the clustering of several samples into a single sequencing lane, and deep-sequencing analyses were performed in triplicate from three independent cell growth, RNA isolation and cDNA library constructions. Raw fastq sequences were obtained from the Illumina Genome Analyzer II (Illumina, San Diego, CA, USA) using the ‘Demultiplex' algorithm in the CASAVA 1.8.2 software (Illumina) that allows the identification of individual samples by ‘index sequences' contained within the adapters and introduced during the adapter ligation and amplification of the samples. miRNAKey was used for data analysis at default settings. The pipeline clips the Illumina 3' adapter sequences from the reads, maps the clipped reads to miRBase and uses the Seq-EM algorithm to estimate the distribution of multiply mapped reads across the observed miRNAs. Sequences <16 bases after adaptor clipping were removed. The read counts obtained were then used for differential expression analysis comparing vector versus Pax3-FOXO1 (V vs PF) or vector versus Pax3 (V vs P3) between control and experimental samples using EBSeq from the R package with a false discovery rate of 5%. We used the default ‘Median Normalization' in EBSeq to make the counts comparable across samples. Target genes for each miRNA were identified either as a result of the indicated PubMed search or using miRTarBase,^29^ which lists experimentally validated direct targets. Several miRNA are expressed in clusters and show similar changes in expression.

a Upregulated by PAX3-FOXO1 and downregulated by PAX3.

b Downregulated by PAX3-FOXO1 and unaffected by PAX3.

c Unaffected by PAX3-FOXO1 and upregulated by PAX3.
